# Color Relation of Metals

**Published:** 1882-09

**Authors:** 


### ARTICLE VII.

### *Color Relation of Metals.*

In a paper on the color relations of copper, nickel, cobalt, iron, maganese, and chromium, lately read before the Chemical Society, Mr. T. Bayley records some remarkable relations between solutions of these metals. It appears that iron, cobalt, and copper form a natural color group, for if solutions of their sulphates are mixed together in the proportions of 20 parts of copper, 7 of iron, and 6 of cobalt, the resulting liquid is free from color, but is grey and partially opaque. It follows from this that a mixture of any two of these elements is complimentary to the third, if the above proportions are maintained. Thus a solution of cobalt (pink) is complementary to a mixture of iron and copper (bluish-green); a solution of iron (yellow) to a mixture of copper and cobalt (violet); and a solution of copper (blue) to a mixture of cobalt and iron (red). But, as Mr. Bayley shows, a solution of copper is exactly complementary to the red reflection from copper, and a polished plate of this metal viewed through a solution of copper salt of a certain thickness, is silver white. As a further consequence it follows that a mixture of iron (7 parts) and cobalt (6 parts) is identical in color with a plate of copper. The resemblance is so striking, that a silver or platinum vessel covered to the proper depth with such a solution, is indistinguishable from copper.

There is a curious fact regarding nickel, also worthy of attention. This metal forms solutions, which can be exactly stimulated by a mixture of iron and copper solutions; but this mixture contains more iron than that which is complementary to colbat. Nickel solutions are almost complementary to colbat solutions, but they transmit an excess of yellow light. Now the atomic weight of nickel is very nearly the mean of the atomic weight of iron and copper, but it is a little lower, that is, nearer to the iron. There is thus a perfect analogy between the atomic weights and the color properties in this case. This analogy is even more general, for Mr. Bayley states that in the case of iron, cobalt, and copper, the mean wave length of the light absorbed is proportional to the atomic weight. The specific chromatic power of the metals varies, being least for copper. The specific chromatic power increases with the affinity of the metal for oxygen. Chromium forms three kinds of salts: pink salts, identical in color with the cobalt salts: blue salts, identical in color with copper salts; and green salts, complimentary to the red salts.

Maganese, in like manner, forms more than one kind of salt. The red salts of cobalt salts and with the red chromium salts. The salts of cromium and manganese, according to the author, are with difficulty attainable in a state of chromatic purity. He thinks that these properties of the metals lead up to some very interesting considerations. —*Chemical Review.*

